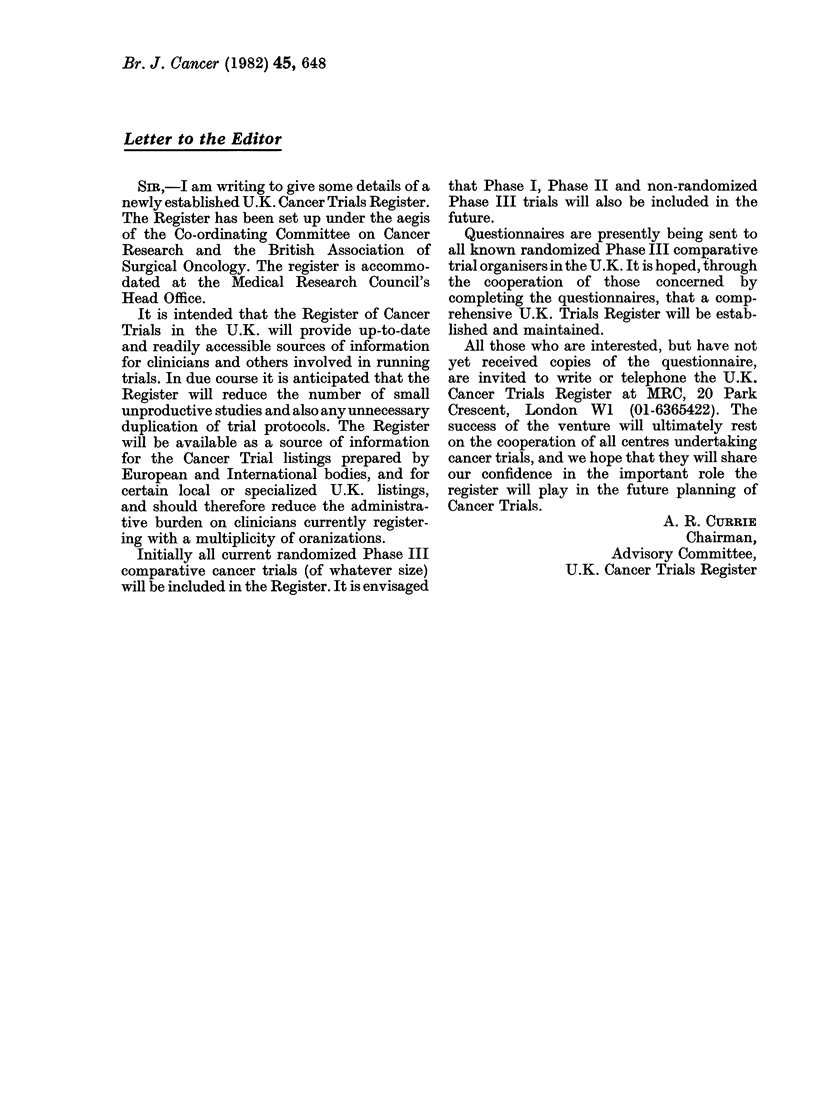# Letters to the Editor

**Published:** 1982-04

**Authors:** A. R. Currie


*Br. J. Cancer* (1982) **45**, 648

### *Letter to the Editor*

SIR,—I am writing to give some details of a newly established U.K. Cancer Trials Register. The Register has been set up under the aegis of the Co-ordinating Committee on Cancer Research and the British Association of Surgical Oncology. The register is accommodated at the Medical Research Council's Head Office.

It is intended that the Register of Cancer Trials in the U.K. will provide up-to-date and readily accessible sources of information for clinicians and others involved in running trials. In due course it is anticipated that the Register will reduce the number of small unproductive studies and also any unnecessary duplication of trial protocols. The Register will be available as a source of information for the Cancer Trial listings prepared by European and International bodies, and for certain local or specialized U.K. listings, and should therefore reduce the administrative burden on clinicians currently registering with a multiplicity of oranizations.

Initially all current randomized Phase III comparative cancer trials (of whatever size) will be included in the Register. It is envisaged that Phase I, Phase II and non-randomized Phase III trials will also be included in the future.

Questionnaires are presently being sent to all known randomized Phase III comparative trial organisers in the U.K. It is hoped, through the cooperation of those concerned by completing the questionnaires, that a comprehensive U.K. Trials Register will be established and maintained.

All those who are interested, but have not yet received copies of the questionnaire, are invited to write or telephone the U.K. Cancer Trials Register at MRC, 20 Park Crescent, London W1 (01-6365422). The success of the venture will ultimately rest on the cooperation of all centres undertaking cancer trials, and we hope that they will share our confidence in the important role the register will play in the future planning of Cancer Trials.

A. R. CURRIE
Chairman,
Advisory Committee,
U.K. Cancer Trials Register